# Efficacy of pulsed electromagnetic field therapy on pain and physical function in patients with non-specific low back pain: a systematic review

**DOI:** 10.1007/s10354-023-01025-5

**Published:** 2023-11-24

**Authors:** Philipp Kull, Mohammad Keilani, Franziska Remer, Richard Crevenna

**Affiliations:** 1 Department of Physical Medicine, Rehabilitation and Occupational Medicine, Medical University of Vienna, Vienna, Austria; 2 Department of Physical Medicine, Rehabilitation and Occupational Medicine, Medical University of Vienna, Waehringer Guertel 18–20, 1090 Vienna, Austria

**Keywords:** PEMF, Pulsed electromagnetic field therapy, Non-specific low back pain, Pain, Physical function, PEMF, Pulsierende Magnetfeldtherapie, Unspezifischer unterer Rückenschmerz, Schmerzen, Funktion

## Abstract

**Introduction:**

Non-specific low back pain is a common and clinically significant condition with substantial socioeconomic implications. Pulsed electromagnetic field (PEMF) therapy has shown benefits in pain reduction and improvement of physical function in patients with pain-associated disorders like osteoarthritis. However, studies had heterogeneous settings. The aim of this study was to assess the effects of PEMF on pain and function on patients with non-specific low back pain.

**Methods:**

A systematic literature search of randomized controlled trials in PubMed, MEDLINE, EMBASE, Cochrane Library, and PEDro was performed (from inception until 15/5/2023). Outcome measures assessed pain and function.

**Results:**

Nine randomized controlled trials with 420 participants (*n* = 420) were included. The studies compared PEMF vs. placebo-PEMF, PEMF and conventional physical therapy vs. conventional physical therapy alone, PEMF and conventional physical therapy vs. placebo-PEMF and conventional physical therapy, PEMF vs. high-intensity laser therapy (HILT) vs. conventional physical therapy, and osteopathic manipulative treatment (OMT) and PEMF vs. PEMF alone vs. placebo-PEMF vs. OMT alone. Five of the nine included studies showed statistically significant pain reduction and improvement in physical function in comparison to their control groups (*p* < 0.05). There was substantial heterogeneity among the groups of the study, with a wide range of duration (10–30 min), treatments per week (2–7/week), applied frequencies (3–50 Hz), and intensities (2mT–150mT). No serious adverse event had been reported in any study. The included studies showed solid methodological quality, with an overall score of 7.2 points according to the PEDro scale.

**Conclusion:**

PEMF therapy seems to be a safe and beneficial treatment option for non-specific low back pain, particularly if used as an addition to conventional physical therapy modalities. Future research should focus on standardized settings including assessment methods, treatment regimens, frequencies, and intensities.

## Abstract (de)

**Hintergrund:**

Der unspezifische unterer Rückenschmerz (Kreuzschmerz, „low back pain“, LBP) ist häufig und klinisch sowie sozioökonomisch höchst relevant. Die pulsierende Magnetfeldtherapie („pulsed electromagnetic field therapy“, PEMF) hat sich bei muskuloskeletalen Schmerzsyndromen (u. a. Kniearthrose) in bis dato sehr heterogenen Studiensettings als wirksam erwiesen. Ziel der vorliegenden systematischen Literaturübersicht war die Darstellung der Effekte von PEMF auf die Parameter Schmerz und Funktion bei Patienten mit unspezifischem LBP.

**Methodik:**

Dazu wurde eine systematische Literatursuche in wissenschaftlichen Datenbanken (PubMed, EMBASE, MEDLINE, Cochrane Library und PEDro) von deren Gründung bis zum 15. Mai 2023 durchgeführt. Es wurden ausschließlich randomisierte kontrollierte klinische Studien eingeschlossen, in denen die Wirkung der PEMF auf die Schmerzintensität und die körperliche Funktion untersucht wurde.

**Ergebnisse:**

Es wurden insgesamt 9 randomisierte kontrollierte Studien mit 420 Teilnehmern (*n* = 420) gefunden. In den Studien erfolgte der Vergleich von PEMF vs. Placebo-PEMF, PEMF und konventionelle physikalische Therapie vs. alleinige konventionelle physikalische Therapie, PEMF und konventionelle physikalische Therapie vs. Placebo-PEMF und konventionelle physikalische Therapie, PEMF vs. High-Intensity-Laser-Therapie (HILT) vs. konventionelle physikalische Therapie und schließlich der Vergleich von osteopathischer Manipulationsbehandlung (OMT) und PEMF vs. alleinige PEMF vs. Placebo-PEMF vs. alleinige OMT. In 5 der 9 inkludierten Studien wurden statistisch signifikante Ergebnisse in der Schmerzreduktion und eine Steigerung der physischen Funktion im Vergleich zur Kontrollgruppe (*p* < 0,05) erzielt. Die eingeschlossenen Studien zeigten eine ausgeprägte Heterogenität in den Therapieregimen (Dauer 10–30 min, Häufigkeit 2‑ bis 7‑mal pro Woche) und den applizierten Frequenzen (3–50 Hz) und Intensitäten (2–150 mT). In keiner der Studien wurde eine ernsthafte Nebenwirkung durch PEMF beschrieben. Der durchschnittliche Wert der methodologischen Qualität betrug 7,2 durch die PEDro-Skala.

**Schlussfolgerung:**

PEMF scheint eine nebenwirkungsarme und effektive Therapie in der Behandlung von unspezifischen Rückenschmerzen zu sein. Vor allem als additive Therapie zu konventionellen physikalischen Maßnahmen konnte eine gute Wirksamkeit gezeigt werden. Weitere wissenschaftliche Untersuchungen sind notwendig, um die Wirksamkeit der PEMF bei unspezifischen Rückenschmerzen zu belegen. Ebenfalls sollte eine Standardisierung der Therapieregime, Frequenzen und Intensitäten angestrebt werden.

## Introduction

Low back pain has a relatively high incidence and prevalence [1]. It affects more than 80% percent of people once in their lifetime [2]. It is not just a major medical problem and the second largest reason for sick leave, with massive impacts on health care systems, but also a huge economic burden with costs of many billions every year [3].

Low back pain is defined as any pain or discomfort between the 12th rib and the gluteal crest, with or without leg pain [1]. The classification of low back pain is difficult because of the varying symptoms and the complex origin of pain. It is often distinguished as acute (less than 6 weeks), subacute (6–12 weeks), and chronic (12 weeks or more) pain, which is an internationally accepted categorization [4].

Most cases are non-specific; only in around 10% cases of low back pain is there a specific cause [5]. Generally, non-specific back pain affects people of all ages [6]. However, it has been determined that office work results in an elevated frequency of employees suffering from non-specific low back pain, because of the sitting position and the continuous computer use [7]. Some other risk factors have also been identified for non-specific low back pain, like awkward postures, bending, and twisting positions for a longer period, such as lifting and carrying heavy weights [8].

There is a wide range of different treatment methods available for non-specific low back pain, both pharmacological and non-pharmacological. Especially a combination of both measures has been recommended as first-line treatment for patients with non-specific low back pain. The advantage of physical therapy modalities is that they are non-invasive and have minor side effects. These include heat, massage, spinal manipulation, acupuncture, ultrasound, electrotherapy, yoga, exercise, behavioral therapy, and many others [9].

A promising physical therapeutic option is pulsed electromagnetic field (PEMF) therapy. PEMFs are slow-frequency electromagnetic currents with an extended range of frequencies without a thermal effect. The mechanisms of PEMF are not completely clarified as yet. However, it has been shown that PEMF increased local cellular activities, oxygen availability, and vasodilation in the tissue in several in vitro studies [10, 11]. It has also been reported that PEMF therapy yields benefits in bone unification, acute pain and chronic relief, postoperative swelling reduction, wound healing, osteoporosis, and fibromyalgia [4, 12–14].

Despite the constantly growing use and scientific investigation of PEMF therapy as a conservative treatment option, the evidence in patients with non-specific low back pain is sparse and still lacking a systematization of its effects. Therefore, this systematic review aimed to search for randomized controlled trials that investigated the effectiveness of PEMF therapy in patients with non-specific back pain for enhancing physical function and reducing pain.

## Methods

### Search strategy

The systematic review was conducted according to the Preferred Reporting Items for Systematic Reviews and Meta-Analysis (PRISMA) statement, which intends to improve the quality of reporting of systematic reviews and meta-analyses [15]. The review protocol was not registered.

A systematic search of the scientific literature published until 15 May 2023 was conducted in the scientific databases PubMed, MEDLINE, EMBASE, Cochrane Library, and PEDro using the search terms and Boolean operators (((pemf) OR (pulsed electromagnetic fields)) OR (pulsed electromagnetic field therapy)) AND (back pain))).

### Inclusion criteria

The inclusion criteria for the studies included in this review followed the PICO(S) (population, intervention, control, and outcome [study design]) model:

- *Population*: patients with non-specific low back pain who underwent PEMF therapy alone or in combination with other physical therapeutic modalities.
- *Intervention*: studies reporting on the influence of PEMF.
- *Control*: studies included a control group of placebo-PEMF alone or combined with conventional physical therapy.
- *Outcome*: studies reporting on the influence of PEMF on pain and physical function with validated assessment instruments.
- *Study design*: randomized controlled trials.

### Exclusion criteria

Studies were excluded for the following reasons:

- Design other than a randomized controlled trial.
- If patients were not excluded in the RCTs for specific reasons of low back pain.
- If the results were not documented with validated assessments for pain like, e.g., visual analog scale (VAS) or numeric rating scale (NRS).
- If the results were not documented with validated assessments for physical function like Oswestry Disability Index (ODI) or Roland Morris Disability Questionnaire (RMDQ).
- Animal studies.
- Full-text articles in languages other than English or German.

### Study selection

All titles and abstracts from the selected databases were screened by two independent reviewers (PK and RF). If the inclusion criteria were met, or if further information was needed to determine whether the inclusion criteria were fulfilled, full-text forms of the studies were read and evaluated. After that, the evaluations of the two reviewers were brought together and discussed. If necessary, a third independent reviewer (RC) was consulted.

### Data extraction

A plan for data extraction from the included studies was based on the consensus of the authors. Extracted information was tabulated and a narrative synthesis was carried out. The following categories are included (Table 2): name of author, year of publication, characteristics of patients, intervention groups, treatment regimen, PEMF frequency, PEMF intensity, assessments, measured timepoints, and results. The results were presented with the corresponding *p*-value.

### Methodological quality assessment

The PEDro scale was assessed for the methodological quality of the studies. It has been reported to be a valid and reliable tool to measure the methodological quality of interventional clinical trials [16, 17]. It consists of 11 items. Details of single items (Table 1) have been published elsewhere [16, 17]. Each item is rated with “yes” or “no” and a total PEDro score is calculated from 0 to 10 by adding the ratings of items 2 to 11. A higher score shows a greater methodological quality. The ratings were assessed by the two authors (PK and RF) independently.

**Table 1:** Methodological quality assessment using the PEDro scale

Study	1	2	3	4	5	6	7	8	9	10	11	Total score
Abdelbasset et al. (2021) [18]	Yes	Yes	Yes	Yes	No	No	Yes	Yes	Yes	Yes	Yes	8
Abdelhalim et al. (2019) [19]	Yes	No	No	Yes	No	No	No	Yes	No	Yes	Yes	4
Alzayed et al. (2020) [20]	Yes	Yes	No	Yes	Yes	No	Yes	No	No	Yes	Yes	6
Auger et al. (2020) [21]	Yes	Yes	Yes	Yes	No	No	Yes	Yes	Yes	Yes	Yes	8
Elshiwi et al. (2019) [22]	Yes	Yes	Yes	Yes	Yes	No	Yes	Yes	Yes	Yes	Yes	9
Krath et al. (2017) [4]	Yes	Yes	Yes	Yes	No	No	No	Yes	Yes	Yes	Yes	7
Lee et al. (2006) [23]	Yes	Yes	No	Yes	Yes	Yes	Yes	Yes	No	Yes	Yes	8
Lisi et al. (2019) [24]	Yes	Yes	Yes	Yes	Yes	No	Yes	No	No	Yes	Yes	7
Yasar et al. (2022) [25]	Yes	Yes	Yes	Yes	No	No	Yes	Yes	Yes	Yes	Yes	8

*1* eligibility criteria (this item is not used to calculate the total score), *2* random allocation, *3* concealed allocation, *4* baseline comparability, *5* participant blinding, *6* therapist blinding, *7* assessor blinding, *8* < 15% dropout, *9* intention-to-treat analysis, *10* between group statistical comparisons, *11* point estimate and variability statistical measure

## Results

**Fig. 1:**
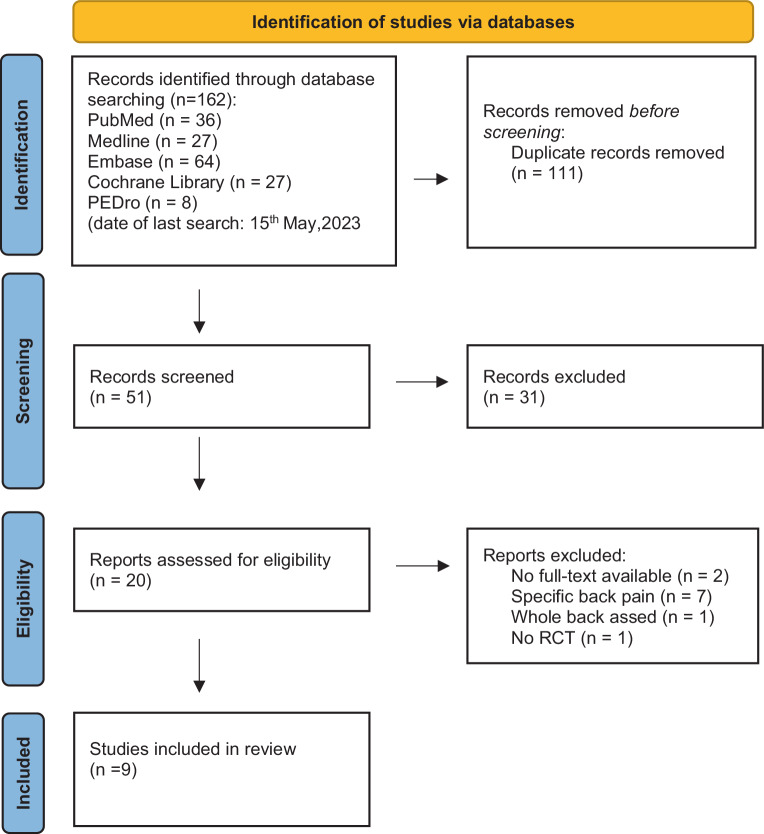
Preferred Reporting Items for Systematic Reviews and Meta-Analyses (PRISMA) flow diagram of the eligibility process. *RCT *randomized controlled trial

A total of 162 articles were found through the systematic database search, which were reduced after duplicate removal and title/abstract reading to 20 full-text articles that were screened for eligibility. An overview of the literature search and selection process is presented in Fig. 1.

Finally, 9 randomized controlled trials were included in the systematic review. Overall, the studies included a total of 420 participants (206 men and 214 women) with a mean average age of 44.6 years. All participants were adults and complained about non-specific low back pain in the included studies [4, 18–25].

There are currently only a small number of quite heterogenous studies that could be included. Therefore, a metanalysis cannot be performed at present.

The inclusion and exclusion criteria varied a little across the studies; the time of duration of back pain was also different. A few studies included participants with acute low back pain for less than 6 weeks, other with chronic low back pain for more than 12 weeks, and some with a mixed duration of pain. All studies only included participants with non-specific back pain. Most of them only included the participants with diagnosed non-specific back pain or excluded them if they had a specific origin of back pain like inflammation or infection, osteoporosis or osteomalacia, spondylolisthesis, history of trauma or major surgery in the lumbar region, major pathologies of the waist or hip, neurological deficits in their lower extremities, or history of malignancy or spinal fracture. Another common exclusion criterion was the presence of a cardiac pacemaker or other electronic implant, which is a contraindication to application of PEMF.

Among the eligible studies, all had at least two groups, with one using a form of PEMF for treatment of non-specific low back pain; one study had three different groups and one even had four groups. However, the groups differed enormously in this review. Two studies compared PEMF vs. a placebo-PEMF, three studies compared PEMF and conventional physical therapy vs. conventional physical therapy alone, two compared PEMF and conventional physical therapy vs. placebo-PEMF and conventional physical therapy, one compared PEMF vs. high-intensity laser (HILT) vs. a conventional exercise therapy and one compared osteopathic manipulative treatment (OMT) and PEMF vs. PEMF alone vs. placebo-PEMF vs. OMT alone.

In general, the studies enrolled the same principles of PEMF therapy, but with different devices and application modes. The frequency used in PEMF therapy ranged from 3 to 50 Hz and the intensity from 2mT to 150mT. Moreover, the treatment regimens showed heterogeneity, so the duration of intervention ranged from 2 weeks to 13 weeks, 2–7 times a week, for 10–30 min once or twice a day. The follow-up also showed some variation and ranged from 2 to 12 weeks. Details on the characteristics of studies, different therapeutic regimens, and outcomes are presented in Table 2.

**Table 2:** Characteristics and main results of the included studies

Study	Demographics	Groups	Treatment regimen	Frequency	Intensity	Measured timepoints	Assessments	Results
Abdelbasset et al. (2021) [18]	*n* = 51	HILT (*n* = 17) vs. PEMF (*n* = 17) vs. control (*n* = 17)	30 min twice a week for 8 consecutive weeks	30 Hz	14mT	Baseline 8 weeks	VAS, ODI, PDI, ROM	PEMF therapy superior to control group after 8 weeks (*p* < 0.05). HILT better than PEMF
	M 34/F 17							
	35.2 years							
Abdelhalim et al. (2019) [19]	*n* = 42	PEMF (*n* = 21) vs. placebo (*n* = 21)	3 sessions per week for 4 weeks	5–10 Hz	2mT	Baseline 4 weeks (after last intervention)	NRS, modified ODI, modified Schober, SF-36	Significant differences in all outcome measure in favor for PEMF group (*p* < 0.05)
	M 28/F 14							
	36.94 years							
Alzayed et al. (2020) [20]	*n* = 42	PEMF + CPT (*n* = 20) vs. placebo-PEMF + CPT (*n* = 22)	20 min per session, 3–5 times per week for 13 weeks	NA	35mT	Baseline 3 weeks 6 weeks 9 weeks 13 weeks	NRS, RMDQ24, PSQI, GPE, DASS-21	No significant difference in assessments between the groups in pain, in physical function at 6.9 and 12 weeks only a significant improvement at week 3 for PEMF (*p* < 0.05)
	M 22/F 20							
	42 years							
Auger et al. (2020) [21]	*n* = 40	OMT + PEMF (*n* = 10) vs. OMT alone (*n* = 10) vs. PEMF alone (*n* = 10) vs. sham PEMF (*n* = 10)	10–20 min 3 times per week for 3 weeks	NA	NA	Baseline 3 weeks	VAS, ODI, SF-12	No significant differences between the groups (*p* > 0.05). OMT + PEMF showed the highest percental decrease in pain and disability
	M 19/F 21							
	25 years							
Elshiwi et al. (2019) [22]	*n* = 50	PEMF + CPT (*n* = 25) vs. placebo-PEMF + CPT (*n* = 25)	20 min per session, 3 sessions per week for 4 weeks	50 Hz	2mT	Baseline 4 weeks	VAS, ODI, ROM	Significant differences in pain and disability for the PEMF group (*p* < 0.05)
	M 25/F 25							
	36.8 years							
Krath et al. (2017) [4]	*n* = 77	PEMF + CPT (*n* = 44) vs. CPT (*n* = 43)	20 min per session, twice per week for 6 weeks	3 Hz	80–150mT	Baseline 6 weeks 12 weeks	ODS, VAS	PEMF + CPT group proved significantly superior to conventional therapy alone (*p* < 0.05)
	M 30/F 47							
	60.4 years							
Lee et al. (2006) [23]	*n* = 36	PEMF (*n* = 17) vs. placebo-PEMF (*n* = 19)	15 min per session three times a week for 3 weeks	5–10 Hz	1.3–2.1 mT	Baseline 1 week 4 weeks (after last intervention)	NRS, ODI	Significant NRS reduction between the groups for the PEMF group (*p* < 0.05). No statistically significant difference in ODI
	M 19/F 17							
	75 years							
Lisi et al. (2019) [24]	*n* = 42	PEMF + CPT (*n* = 23) vs. placebo-PEMF + CPT (*n* = 19)	30 min twice per day for 6 weeks, after that 30 min once a day for 6 weeks	NA	NA	Baseline 6 weeks 12 weeks	NRS, ODI	Significant improvements in ODI at 6 and 12 weeks in the experimental group (*p* < 0.05). Pain just significantly reduced at 6 weeks (*p* < 0.05)
	M 18/F 24							
	35 years							
Yasar et al. (2022) [25]	*n* = 40	PEMF + CPT (*n* = 20) vs. ICF + CPT (*n* = 20)	20 min per session, 5 days a week, for 2 weeks	50 Hz	2.5mT	Baseline 2 weeks 8 weeks	NRS, RMDQ, EuroQol, FtF	Improvement in both groups. No significant differences between groups RMDQ, NRS (*p* > 0.05)
	M 11/F 29							
	55.7 years							

*M* male, *F* female, *PEMF* pulsed electromagnetic field, *HILT* high-intensity laser therapy, *Hz* hertz, *mT* millitesla, *OMT* osteopathic manipulative treatment, *ICF* interference current, *CPT* conventional physical therapy, *VAS* visual analog scale, *NRS* numeric rating scale, *ODI* Oswestry Disability Index, *RMDQ* Roland Morris Disability Questionnaire, *PDI* Pain Disability Index, *ROM* range of motion, *PSQI* Pittsburgh Sleep Quality Index, *GPE* global perceived effect, *DASS-21* Depression Anxiety Stress Scale, *SF-12* Short Form 12, *SF-36* Short Form 36, *EQ-5D* EuroQol—Health-related Quality of Life Questionnaire, *FtF* fingertip-to-floor

### Outcomes of interest

#### Pain

All included RCTs reported outcomes of pain with VAS or NRS. Five studies used the NRS and four studies the VAS. All studies documented at least a reduction in pain in the intervention group. Statistically significant pain reduction compared to the control group was reported in five of the nine studies (*p* < 0.05 in each study). In two studies there was a significant difference at some measured timepoints. Two studies showed no significant pain reduction in the comparison of intervention and control groups at all measured timepoints.

#### Physical function

Physical function was assessed in all of the RCTs, reported with the ODI or a modified form of the ODI in seven studies, and in two studies with the RMDQ. The results of quantification of the patients’ function showed a statistically higher improvement in the group with using PEMF in the comparison to control groups in five studies (*p* < 0.05 in each study). In one study, a statistically significant difference was only found at one of the four measured timepoints. Three studies reported no significant difference in assessment of physical function between the experimental and control groups.

### Methodological quality assessment

The mean score of the PEDro scale for methodological quality of the included studies was 7.2 (range 4–9) out of 10 points. A common methodological limitation was the blinding of subjects, assessors, and therapists, which was not performed in all studies with controlled groups that received conventional physical therapy. Only one study with PEDro score 5 or less had been included, and it is considered to be of low quality. The results of the evaluation of methodological quality of the included literature are shown in Table 1.

## Discussion

The main finding of this systematic review is that PEMF seems to be a beneficial therapy for pain relief and enhancing physical function in patients with non-specific low back pain. Especially when added to other conventional physical therapies, PEMF was shown to have some additional effect in the treatment of patients with non-specific low back pain.

In the study of Krath et al., where 88 patients (*n* = 88) received either a conventional non-invasive treatment with physiotherapy or a combination of conventional non-invasive treatment plus PEMF for 6 weeks, a significant reduction of pain and improvement in the ODI compared to the control group at the 6‑week follow-up and also at the 12-week follow-up was shown [4]. Similar results were reported by Elshiwi et al.: in their study with 50 patients, the control group received conventional physical therapy plus placebo-PEMF and the experimental group the same conventional physical therapy and PEMF, and the authors presented significant differences between the groups [22]. In another study with comparable groups there was only one significant result measured out of four measured timepoints [20]. In the study from Lisi et al., where PEMF with conventional therapy was also compared to placebo-PEMF and conventional therapy, there was just a significant difference found in pain reduction after 6 weeks, but not after 12 weeks [24]. This could be explained by the fact that the PEMF therapy was applied with a home device and the use and regularity could not be controlled for exactly. Often the compliance of home therapy is reduced after the first relief of symptoms [26].

In the comparison of PEMF and placebo-PEMF, the two studies reported conflicting results: the post-treatment comparison from Abdelhalim et al. showed a significant difference in all outcome measures in favor of the experimental group [19], in contrast to Lee et al., where only a difference was found in pain reduction but not in physical function [23].

Previous studies have shown that improvements in pain and function for non-specific low back pain patients are often independent and that the recovery of physical function is a more important outcome [27, 28].

In the studies from Abdelbasset et al., Auger et al., and Yasar et al., another intervention was investigated as well [19, 21, 25]. Abdelbasset et al. used HILT, PEMF, and a control group. A significant difference was noted for the PEMF group compared to the control group, but the HILT group showed greater reduction and improvement than the PEMF group.

HILT, a special form of low-level laser therapy, is well known in the management of different musculoskeletal pain disorders [29, 30]. For low back pain, HILT has shown better improvement compared to ultrasound [31].

In the study of Auger et al. with four different groups, the authors compared PEMF with osteopathic manipulative treatment (OMT), which has been shown to be an effective treatment for low back pain [32, 33]. No statistical significance between the groups was reported because of the small number of participants, with 10 in each group [21]. Nevertheless, the best reduction in this study was reported for the PEMF and OMT group.

Yasar et al. measured the difference between PEMF and conventional physical therapy and interference current (IFC) and conventional physical therapy. There was no significant difference related to the outcome parameters reported in the study [25]. However, IFC is a well-established and frequently used evidence-based treatment for low back pain, which also implies the effectiveness of PEMF in the treatment of low back pain [34].

As described, there was substantial heterogeneity among the groups of the studies, which could explain the different results. Also, just three of the studies used the same PEMF device [18, 19, 21]. Moreover, there was a wide range of applied parameters: the frequency between 3 and 50 Hz. Generally, low frequencies such as those used in the present studies are more often used and recommended by the World Health Organization (WHO) [35]. In addition, the intensities used in the individual studies differed between 2mT and 150mT. It seems that higher intensities would result in better pain reduction and enhancement of physical function, as applied by Krath et al. and Abdelbasset et al. [4, 18].

Prior work which included many different etiologies of back pain, including specific ones like discogenic lumbar radiculopathy or failed back surgery syndrome, has also reported conflicting outcomes, especially for physical function. On the one hand, Sun et al. reported that PEMF did not improve physical function compared to the control group [36]. They included 14 studies and also performed a quantitative analysis. On the other hand, a smaller study from Andrade et al. which included five studies showed an improvement in physical function through PEMF [37]. Concerning reduction of the symptom pain, both studies showed similar effectiveness.

The results of pain reduction by PEMF in patients with lower back pain can be usefully compared to reports on the effects of non-steroidal anti-inflammatory drugs (NSAIDs): the effectiveness of valdecoxib and eterocoxib on lower back pain in randomized, double-blind, placebo-controlled trials was similar to the pain reduction in the study by Lee et al. through the use of PEMF [23, 38, 39].

The mechanism by which PEMF reduces pain is unclear. Several explanations have been put forward to explain its analgesic effects, including subsequent muscle relaxation through hyperpolarization at the motor endplate, depolarization of nociceptive C‑fibers, and stimulation of chondrogenesis [40, 41]. A reduction in inflammatory cytokines and promoted tendon healing could also be found in an animal study [42]. PEMF with frequencies under 60 Hz were found to affect cell behavior by increasing transcription and DNA synthesis [43, 44].

The evidence situation is also not entirely clear for other pain disorders. In the study by Trock et al., a pain reduction in osteoarthritis reported. These authors showed a significant pain reduction in patients with cervical facet osteoarthritis and knee osteoarthritis, which was also confirmed in the follow-up, when the therapy was used alone [40]. On the other hand, a meta-analysis by McCarty et al. revealed that PEMF therapy should be used just as a complementary treatment rather than alone for knee osteoarthritis patients [45].

In the nine trials included in this review, no serious adverse treatment effects were reported. Only in the study by Lisi et al. was a mild event reported in the intervention group, but also in the sham group, through the non-supervised use of the home device [24]. Thus, immediate adverse effects of PEMF therapy are rare. PEMF was also well tolerated by the patients and showed a high degree of compliance in all included studies.

The limitations of the present systematic review are mainly related to the individual limitations of the included studies, which are principally due to the small number of participants and the high heterogeneity of the PEMF interventions, controlled therapies, and treatment regimes. Moreover, the intervention periods were short and there was no long-term follow-up in any of the studies. The main limitation of this systematic review is the small number of studies that could be included. In addition, only studies in English or German were included. However, previous work demonstrated that restriction to the English language in systematic review does not cause additional bias [46, 47].

## Conclusion

The results of the present systematic review suggest that the use of PEMF for patients with non-specific low back pain is beneficial in terms of pain reduction and enhancement of physical function, particularly if used as an addition to conventional physical therapy modalities. It has also been shown that PEMF is a safe therapy for the treatment of non-specific low back pain. Further high-quality studies with larger sample sizes and standardized protocols are necessary. The studies should also focus on determining the optimal parameters of frequency and intensity to advance PEMF application for all pain disorders.
